# A Syndrome-Based Surveillance System for Infectious Diseases Among Asylum Seekers in Austrian Reception Centers, 2015-2018: Analysis of Reported Data

**DOI:** 10.2196/11465

**Published:** 2019-02-27

**Authors:** Ziad El-Khatib, Karin Taus, Lukas Richter, Franz Allerberger, Daniela Schmid

**Affiliations:** 1 Department of Surveillance and Infectious Disease Epidemiology Institute of Medical Microbiology and Hygiene Austrian Agency for Health and Food Safety Vienna Austria; 2 Department of Public Health Sciences Karolinska Institutet Stockholm Sweden

**Keywords:** Austria, refugee health, asylum seekers, syndrome surveillance system, mass health monitoring, refugees, population surveillance, public health surveillance, epidemiological monitoring

## Abstract

**Background:**

Austria has been among the main European countries hosting incoming asylum seekers since 2015. Consequently, there was an urgent need to predict any public health threats associated with the arriving asylum seekers. The Department of Surveillance and Infectious Disease Epidemiology at the Austrian Agency for Health and Food Safety (AGES) was mandated to implement a national syndrome-based surveillance system in the 7 reception centers by the Austrian Ministry of Interior and Ministry of Health.

**Objective:**

We aimed to analyze the occurrence and spread of infectious diseases among asylum seekers using data reported by reception centers through the syndrome-based surveillance system from September 2015 through February 2018.

**Methods:**

We deployed a daily data collection system for 13 syndromes: rash with fever; rash without fever; acute upper respiratory tract infection; acute lower respiratory tract infection; meningitis or encephalitis; fever and bleeding; nonbloody gastroenteritis or watery diarrhea; bloody diarrhea; acute jaundice; skin, soft tissue, or bone abnormalities; acute flaccid paralysis; high fever with no other signs; and unexplained death. General practitioners, the first professionals to consult for health problems at reception centers in Austria, sent the tally sheets on identified syndromes daily to the AGES.

**Results:**

We identified a total of 2914 cases, presenting 8 of the 13 syndromes. A total of 405 signals were triggered, and 6.4% (26/405) of them generated alerts. Suspected acute upper respiratory tract infection (1470/2914, 50.45% of cases), rash without fever (1174/2914, 40.29% of cases), suspected acute lower respiratory tract infection (159/2914, 5.46% of cases), watery diarrhea (73/2914, 2.51% of cases), and skin, soft tissue, or bone abnormalities (32/2914, 1.10% of cases) were the top 5 syndromes.

**Conclusions:**

The cooperation of the AGES with reception center health care staff, supported by the 2 involved ministries, was shown to be useful for syndromic surveillance of infectious diseases among asylum seekers. None of the identified alerts escalated to an outbreak.

## Introduction

### Background

Since 2015, over 1.5 million refugees and asylum seekers have reached Europe [1], where Austria, Sweden, and Hungary were the top 3 countries per capita in hosting this vulnerable population [1]. The majority of these asylum seekers came from Syria (49%), Afghanistan (21%), and Iraq (9%) [2,3].

The asylum seekers entered Austria largely through the Balkan corridor. The countries they travel through include, in the order, are Turkey, Greece, the Former Yugoslav Republic of Macedonia, Serbia, Croatia, Slovenia, and finally Austria [2,4], during a period ranging between 1 and 7 months [5]. Among the top 5 countries of origin of the asylum seekers who entered Austria in 2015-2016, over half of those were from Afghanistan and Syria, followed by Iraq, Iran, and Pakistan [6]. On behalf of the Republic of Austria, since 2012, a medical organization, called in German *Organisation für Regie und Spezialaufträge* (ORS Service AG, Zurich, Switzerland), has looked after all asylum seekers who are in federal care, including the operation of 3 permanent reception centers (RCs) for asylum seekers. Health services, including physical examination at arrival and need-based health care, are provided in the RCs by a physician and a nurse.

A refugee population is considered as a vulnerable group due to their living conditions during migration and their psychological stress, combined with limited access to sanitation, health services, and nutritious food [4]. This places asylum seekers, especially children and women, at a higher risk of acquiring communicable diseases during their migration in transit and in host countries [7]. A systematic evaluation of health conditions of migrants at the RCs will inform health care service providers [8]. In July 2015, following a recommendation of the Austrian Agency for Health and Food Safety (AGES), the Austrian Ministry of Health together with the Ministry of Interior decided to implement a syndrome-based surveillance system (SbSS) in all of the 7 national RCs for asylum seekers. The AGES implemented this SbSS in 3 phases, starting in August 2015. Phase 1 was preparatory, which included the selection of the RCs and collection of information on the number of beds in the RCs, applied hygiene measures, and procedure of the health status assessment of asylum seekers at their arrival at the center. We assessed the risk for epidemic-prone infectious diseases existing in the country of migrant origin, prevailing in transit countries, present in the host country, and favored by the immunization status, as recommended by the European Centre for Disease Prevention and Control (ECDC) [9]. Finally, we prepared a protocol on the SbSS. Phase 2 was piloting the SbSS in 2 of the 7 RCs in September 2015. These 2 were located in 2 of the 9 Austrian provinces (Lower Austria and Tyrol). Phase 3 included the implementation of the SbSS. In November 2015, the ECDC published a report based on expert opinion on the public health needs of asylum seekers at the southern and southeastern borders of the European Union (EU) [4]. According to this document, the public health measures to be considered included (1) disease screening, (2) vaccination, (3) access to health care, free of charge, general hygiene measures, and prevention of overcrowding in RCs, and (4) implementation of an SbSS [10].

Overall, SbSS is a reliable method for public health surveillance that has been used in different countries and contexts. It provides timely information and supports suitable public health responses [11]. According to EU member state experiences [4], the following syndromes were suggested: (1) respiratory tract disease, (2) suspected pulmonary tuberculosis, (3) bloody diarrhea, (4) watery diarrhea, (5) fever and rash, (6) meningitis or encephalitis, or encephalopathy or delirium, (7) lymphadenitis with fever, (8) botulism-like illness, (9) sepsis or unexplained shock, (10) hemorrhagic illness, (11) acute jaundice, (12) parasitic skin infection, and (13) unexplained death [4,10,12].

### Objective

The main objective of the Austrian SbSS was to enhance early detection of single cases or outbreaks of infectious diseases that require an assessment to initiate and guide appropriate public health measures in order to prevent spread of these diseases among the migrant population and the host population. To our knowledge, the use of the SbSS method among asylum seekers in the EU has not been thoroughly documented during the current refugee crisis. In this paper, we present and describe the results of using a national SbSS for asylum seekers in Austrian RCs.

## Methods

### Description of the Syndromic Surveillance System

#### Population and Syndromes Under Surveillance

The population under surveillance comprised asylum seekers hosted by the 7 Austrian RCs, which were located in 7 of the 9 Austrian provinces (Table 1). The SbSS was rolled out in these 7 centers one after another, 6 between September and December 2015, and the last 1 in August 2016. Table 1 gives the RCs by province and dates of implementation of the SbSS. The longest period of surveillance was in the permanent RC, which is located in the province of Lower Austria (RC1 east) for a duration up to 2.5 years.

The surveillance included 13 syndromes (Table 2), which we selected according to the infectious disease risks assessed specifically for asylum seekers in Austria and to the ECDC’s assessment for risks related to asylum seekers in Europe [13-15]. The syndromes were rash with fever; rash without fever; suspected acute upper respiratory tract infection (URTI); suspected acute lower respiratory tract infection (LRTI); meningitis or encephalitis; fever and bleeding; non–bloody gastroenteritis or watery diarrhea; bloody diarrhea; acute jaundice; skin, soft tissue, or bone abnormalities; acute flaccid paralysis; high fever with no other signs; and unexplained death. Table 2 presents the 13 syndromes by name and gives the definition, underlying target diseases, and relevant public health actions.

**Table 1:** Reception centers in Austria by duration of syndrome-based surveillance in place and number of beds.

Province (geographical location inside Austria)	Reception center	Surveillance start date	Duration of surveillance (days)	Beds (N)
Lower Austria	RC1 (east)	September 2015	890	1800
Tyrol	RC2 (west)	September 2015	873	200
Upper Austria	RC3 (north)	October 2015	852	180
Carinthia	RC4 (south)	December 2015	804	200
Vienna	RC5 (east)	December 2015	797	150
Styria	RC6 (south)	December 2015	787	150
Salzburg	RC7 (west)	August 2016	538	160

#### Case Finding and Case Reporting

In Austrian RCs, health care staff, including general practitioners and nurses, are the first professionals to consult for health problems of asylum seekers. The health care staff assessed the presence of syndromes, as defined in Table 2, during the medical entry examination (active case finding) and any other consultation required by asylum seekers during their stay at the RC (passive case finding). The RC’s SbSS focal person accordingly filled in a tally sheet, which listed the 13 syndromes with their clinical definition by age group (0-4, 5-14, 15-44, 45-64, ≥65 years) and was asked to send it on a daily basis to the SbSS project manager at the AGES by fax, email, or an online data entry form.

#### Quality Indicators Used for Syndromic Surveillance System Evaluation

We defined completeness and timeliness as a completed tally sheet (including “zero” reporting) sent within 24 hours of detection to the AGES.

### Data Analysis

**Figure 1:**
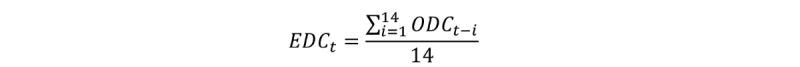
Formula for the expected daily cases (EDCs) at day *t* defined as the 1-sided moving average of the syndrome-specific observed daily cases (ODCs) of the previous 14 days.

We adapted and modified the model for signal and alert generation as described by Napoli et al and applied in refugee RCs in Italy [16].We determined the number of observed (reported) daily cases for each syndrome (syndrome-specific ODCs) and RC, including zero reporting. By calculating the 1-sided moving average of the syndrome-specific ODCs of the previous 14 days with the formula shown in Figure 1, we determined the expected daily cases (EDCs). We created a threshold for the expected daily cases (ETH) by adding 2 times the standard deviation to the EDCs (ie, ETH_t_ = EDCs_t_ + 2 × SD_t_). We measured the ODCs daily against the ETH for each syndrome. A signal was generated when the number of syndrome-specific ODCs exceeded the ETH for the syndrome, and an alert was defined as signals occurring over 2 consecutive days. We analyzed the data and generated outputs (syndrome-specific number of signals and alerts) automatically on a daily, weekly, and monthly basis using R [17]. We shared the weekly reports with the Federal Ministry of Health and the monthly reports with the contact person at each RC. In case the RCs did not send the daily report, the manager of the SbSS called the contact person at the respective RC to follow up on the status of the report. In case of a signal, the SbSS manager at the AGES immediately called the respective RC contact person to clarify the underlying diseases and to verify the cluster of cases. In case of an alert, the AGES immediately informed the Ministry of Health.

### Ethics and Informed Consent

The SbSS was conducted as part of the health care services offered by the Austrian Ministry of Interior, in collaboration with the Ministry of Health and the Department of Surveillance and Infectious Disease Epidemiology at the AGES. As data were collected in an anonymous fashion, there was no need for ethical clearance.

**Table 2:** List of syndromes surveilled and their definition, target diseases, and public health action^a^.

Name of syndrome	Definition of syndrome	Target disease	Public health action or measures
Rash with fever	Temperature ≥38.0°C and generalized rash of any nature	Measles, rubella, varicella, smallpox, louse-borne diseases (relapsing fever due to *Borrelia recurrentis*, trench fever due to *Bartonella quintana*, epidemic typhus due to *Rickettsia prowazekii*).	Outbreak confirmation and investigation, contact tracing, isolation, vaccination
Rash without fever	N/A^b^	Scabies.	N/A
Suspected acute upper respiratory tract infection	Fever, cough, sore throat, runny nose	Pharyngitis, tonsillitis caused by adenovirus, rhinovirus, respiratory syncytial virus, influenza, parainfluenza.	N/A
Suspected acute lower respiratory tract infection with fever	Temperature ≥38.0°C and at least one of the following signs or symptoms: breathing difficulties; chest rales or increased respiratory rate	Tracheitis, bronchitis, pneumonia, bronchopneumonia, or bronchiolitis, including those caused by, for example, adenovirus, streptococci, pneumococci, *Mycoplasma* *,* *Legionella*.	Outbreak investigation in case of clustering of cases
Meningitis or encephalitis	Temperature ≥38.0°C and at least one of the following signs or symptoms: severe, persistent headache; neck stiffness; altered consciousness; altered mental status; confusion; delirium; or disorientation	Bacterial, viral, fungal, or other infectious meningitis or encephalitis. This could be caused by meningococci, *Haemophilus influenzae*, pneumococci, *Listeria*, *Leptospira*, *Mycobacterium tuberculosis*, *Treponema pallidum*, enteroviruses, poliovirus, measles virus, mumps virus, rubella virus, influenza virus, West Nile virus, other arboviruses.	Outbreak confirmation and investigation, contact tracing, isolation
Fever and bleeding	Temperature ≥38.0°C and at least one of the following signs or symptoms: petechial rash with any purpuric areas; hemorrhagic exanthema; hematuria; conjunctival hemorrhage; gingival bleeding; epistaxis; bloody diarrhea; unexplained bleeding from other sites; or clinical suspicion of a viral hemorrhagic illness	Hemorrhagic fevers due to infectious disease agents. These could include yellow fever, dengue, or Crimean-Congo hemorrhagic fever and other arboviral diseases, Ebola.	Contact tracing, isolation
Non–bloody watery diarrhea	≥3 watery stools per day, nausea, vomiting	Gastroenteritis caused by norovirus, rotavirus, bacterial toxins. *Campylobacter*, *Salmonella*, *Escherichia coli*, *Yersinia*, *Vibrio cholerae*.	Outbreak investigation for source and vehicle, and control in case of clustering of cases
Bloody diarrhea	Red blood in the stool	Amoebic dysentery, *Shigella*, *Campylobacter*, verotoxin-producing *E coli*.	Outbreak investigation for source and vehicle, and control in case of clustering of cases
Acute jaundice	Acute onset of jaundice and at least one of the following signs or symptoms: temperature ≥38.0°C; malaise or hepatomegaly	Acute viral hepatitis A and E; other hepatitis.	Outbreak investigation for source and vehicle
Skin, soft tissue, or bone abnormalities	Skin or soft tissue lesions, ulceration, inflammation	Cutaneous diphtheria, cutaneous tuberculosis, cutaneous leishmaniasis, bacterial wound infection.	N/A
Acute flaccid paralysis	Person under the age of 15 years with acute flaccid nonsymmetrical paralysis	Acute flaccid paralysis, or paralytic or poliomyelitis.	Contact tracing, immunization
High fever with no other signs	High fever up to 40°C or more, persisting, intermittent, long-lasting	Typhoid fever; malaria, or visceral leishmaniasis.	N/A
Unexplained death	N/A	N/A	N/A

^a^Sources: European Centre for Disease Prevention and Control [4,10].

^b^N/A: not applicable.

## Results

### Reports of Cases, Signals, and Alerts

During the period of September 2015 through February 2018, a total of 2914 cases, showing 8 of the 13 syndromes surveilled, were reported. The majority of patients were aged between 15 and 44 years (2179/2913, 74.80%), followed by children aged between 5 and 14 years (369/2913, 12.67%) and under 5 years (311/2913, 10.68%; Table 3).

**Figure 2:**
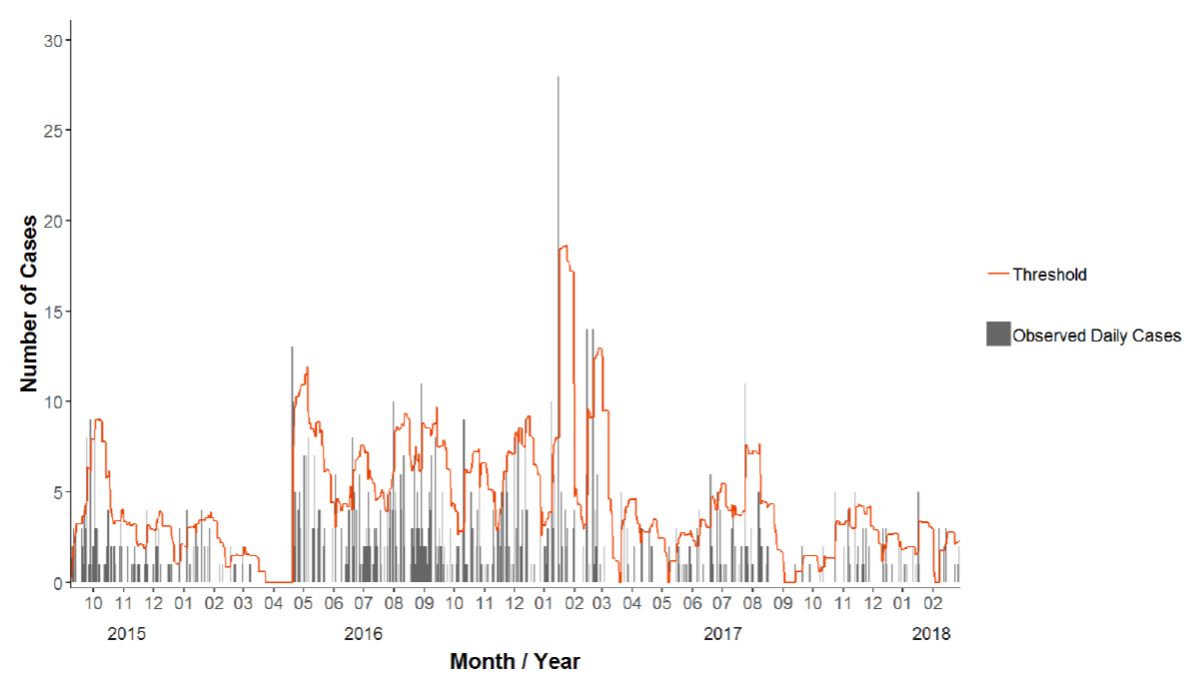
Observed daily cases of rash without fever, illustrated by gray bars, and threshold of the expected daily cases, shown by the orange line (n=151), 2015-2018, Austria.

The top 5 syndromes by number of cases were (1) suspected URTI (1470/2914, 50.45% or cases), (2) rash without fever (entirely due to scabies; 1174/2914, 40.29% of cases), (3) suspected acute LRTI with fever (159/2914, 5.46% of cases), (4) watery diarrhea (73/2914, 2.51% of cases), and (5) skin, soft tissue, or bone abnormalities (32/2914, 1.10% of cases; Table 4). Figure 2 shows a plot of the data output for the example of the number of ODCs of scabies.

**Figure 3:**
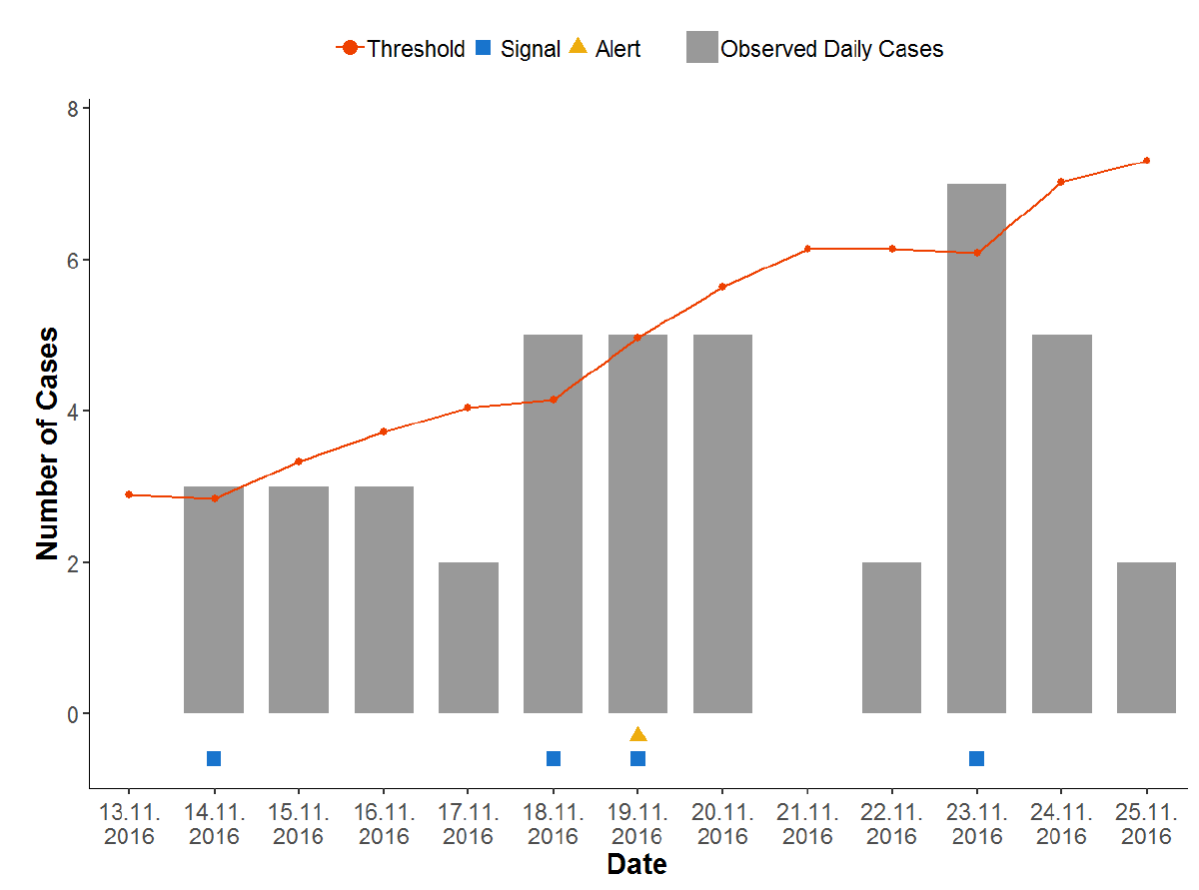
Selected 2 weeks of surveillance, during November 13-25, 2016, illustrating the observed daily cases of rash without fever by bars, threshold of the expected daily cases by orange line, the signals by blue squares (n=4; at November 14, 18, 19, and 23, 2016), and the alerts by a yellow triangle (n=1; at November 19, 2016).

**Figure 4:**
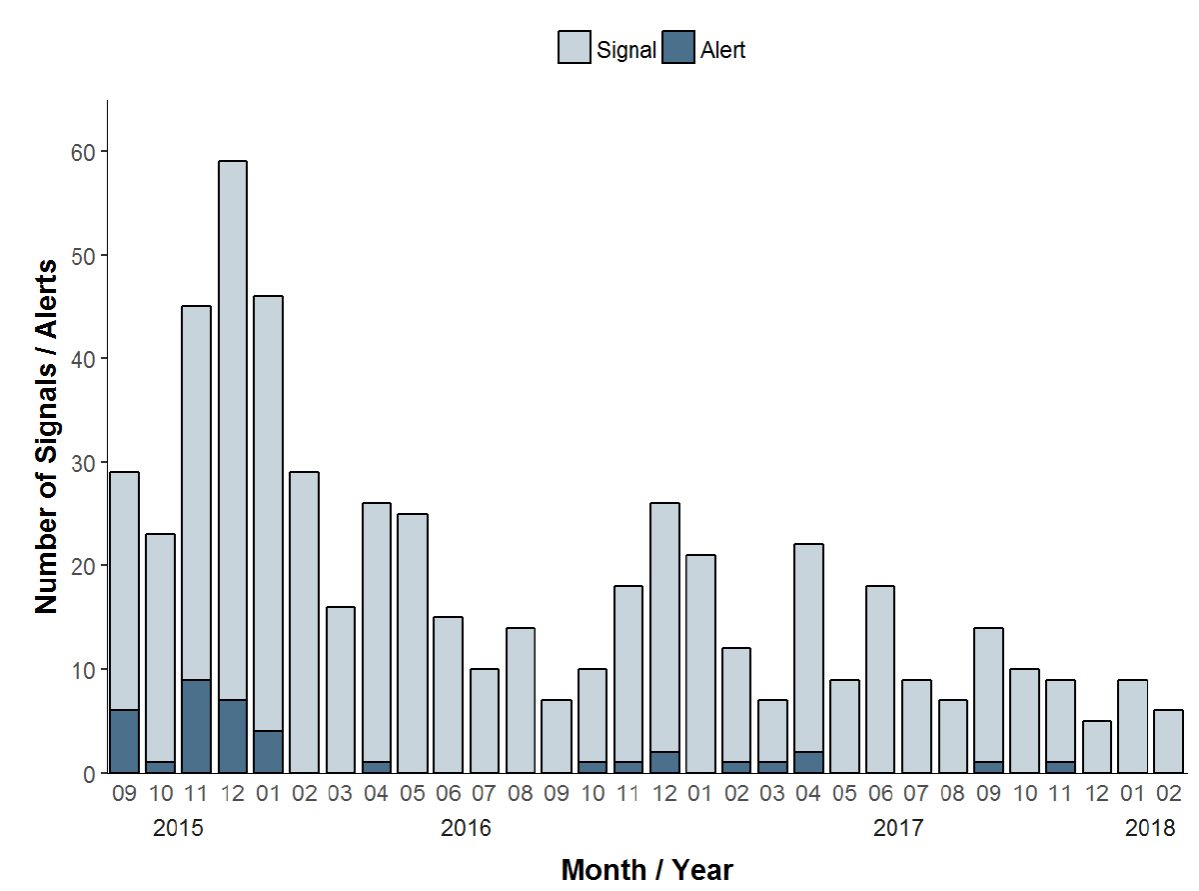
Numbers of signals and alerts generated by the syndrome-based surveillance system for all syndromes, per month, September 2015-February 2018, Austria.

Among the total 405 triggered signals, the predominating signal-causing syndromes were suspected URTI (151/405, 37.3%), rash without fever (142/405, 35.1%), and suspected LRTI (67/405, 16.5%). Of the 405 signals, 26 (6.4%) triggered an alert, which were due to suspected acute URTI (10/26, 39%), rash without fever (10/26, 39%), suspected acute LRTI (4/26, 15%), and skin, soft tissue, or bone abnormalities (2/26, 8%; see Table 5). Figure 3 shows a plot of the data output for the example of the number of signals and alerts for scabies. Figure 4 shows the monthly number of signals and alerts for all syndromes during September 2015 through February 2018.

### Laboratory Diagnostics of the Case-Patients

Of the 1470 case-patients with URTI, 21 of 30 patients (70%) with influenza-like illness tested positive for influenza during the 2015-2016 influenza season. Among asylum seekers with acute LRTI with fever and cough, 3 cases were diagnosed as pulmonary tuberculosis. Of the 73 cases of watery diarrhea, 3 cases were culture-confirmed shigellosis. As the others had short-term diarrhea, the causative pathogen was not identified. One case of malaria was identified among those with high fever with no other signs.

### Quality Indicators

Among the RCs, only RC1 and RC2, with permanent health care staff, sent tally sheets on syndromes daily within 24 hours. The remaining RCs sent the sheets whenever the health care service was delivered at the site.

**Table 3:** Number of cases of each syndrome in total and by age group recorded by the syndrome-based surveillance system, Austria, September 2015-February 2018.

Name of syndrome	All ages^a^	Age groups (years)				
		0-4	5-14	15-44	45-64	≥65
Suspected acute URTI^b^, n (%)	1469 (50.43)	235 (75.6)	291 (78.9)	911 (41.81)	31 (60)	1 (50)
Rash without fever, n (%)	1174 (40.30)	20 (6.4)	55 (14.9)	1095 (50.25)	4 (8)	0 (0)
Suspected acute LRTI^c^ with fever, n (%)	159 (5.46)	16 (5.1)	12 (3.3)	115 (5.28)	16 (31)	0 (0)
Watery diarrhea, n (%)	73 (2.51)	34 (10.9)	11 (3.0)	27 (1.24)	1 (2)	0 (0)
Skin, soft tissue, or bone abnormalities, n (%)	32 (1.10)	1 (0.03)	0 (0)	30 (1.38)	0 (0)	1 (50)
Rash with fever, n (%)	4 (0.14)	4 (0.13)	0 (0)	0 (0)	0 (0)	0 (0)
Meningitis or encephalitis, n (%)	0 (0)	0 (0)	0 (0)	0 (0)	0 (0)	0 (0)
High fever (≥39°C) with no other signs, n (%)	1 (0.03)	0 (0)	0 (0)	1 (0.05)	0 (0)	0 (0)
Unexplained death, n (%)	1 (0.03)	1 (0.3)	0 (0)	0 (0)	0 (0)	0 (0)
Total (N)	2913	311	369	2179	52	2

^a^One case-patient had missing information on age.

^b^URTI: upper respiratory tract infection.

^c^LRTI: lower respiratory tract infection.

**Table 4:** Number of cases of each syndrome, in total and stratified by reception center (RC; RC1-RC7), recorded by the syndrome-based surveillance system, Austria, September 2015-February 2018.

Name of syndrome	All RCs	RC (by geographical location in Austria)						
		RC1 (east)	RC2 (west)	RC3 (north)	RC4 (south)	RC5 (east)	RC6 (south)	RC7 (west)
Start date	—	September 2015	September 2015	October 2015	December 2015	December 2015	December 2015	April 2016
Suspected acute URTI^a^, n (%)	1470 (50.45)	722 (38.16)	127 (70.6)	250 (71.4)	240 (83.0)	28 (39)	64 (73)	39 (74)
Rash without fever, n (%)	1174 (40.29)	1031 (54.49)	38 (21.1)	55 (15.7)	38 (13.2)	6 (8)	0 (0)	6 (11)
Suspected acute LRTI^b^ with fever, n (%)	159 (5.46)	84 (4.44)	5 (2.8)	12 (3.4)	4 (1.4)	26 (36)	22 (25)	6 (11)
Watery diarrhea, n (%)	73 (2.51)	29 (1.53)	10 (5.6)	30 (8.6)	2 (0.7)	8 (11)	2 (2)	2 (4)
Skin, soft tissue, or bone abnormalities, n (%)	32 (1.10)	24 (1.27)	0 (0)	0 (0)	5 (1.7)	3 (4)	0 (0)	0 (0)
Skin rash with fever, n (%)	4 (0.14)	0 (0)	0 (0)	3 (0.9)	0 (0)	1 (1)	0 (0)	0 (0)
Meningitis or encephalitis, n (%)	0 (0)	0 (0)	0 (0)	0 (0)	0 (0)	0 (0)	0 (0)	0 (0)
High fever with no other signs, n (%)	1 (0.03)	1 (0.05)	0 (0)	0 (0)	0 (0)	0 (0)	0 (0)	0 (0)
Unexplained death, n (%)	1 (0.03)	1 (0.05)	0 (0)	0 (0)	0 (0)	0 (0)	0 (0)	0 (0)
Total (N)	2914	1892	180	350	289	72	88	53

^a^URTI: upper respiratory tract infection.

^b^LRTI: lower respiratory tract infection.

**Table 5:** Number of signals and alerts for each syndrome recorded by the syndrome-based surveillance system, Austria, September 2015-February 2018.

Name of syndrome	Signals	Alerts
Suspected acute URTI^a^, n (%)	151 (37.3)	10 (39)
Rash without fever, n (%)	142 (35.1)	10 (39)
Suspected acute LRTI^b^ with fever, n (%)	67 (16.5)	4 (15)
Watery diarrhea, n (%)	27 (6.7)	0 (0)
Skin, soft tissue, or bone abnormalities, n (%)	14 (3.5)	2 (8)
Rash with fever, n (%)	2 (0.5)	0 (0)
High fever (≥39°C) with no other signs, n (%)	1 (0.3)	0 (0)
Unexplained death, n (%)	1 (0.3)	0 (0)
Total (N)	405	26

^a^URTI: upper respiratory tract infection.

^b^LRTI: lower respiratory tract infection.

## Discussion

### Principal Findings

This was the first attempt to use an SbSS in Austria during an unusual influx of asylum-seeking refugees, facing harsh travelling conditions, since World War II. During the total surveillance period from September 2015 to February 2018, more than 400 statistical signals were triggered, caused by 5 predominating syndromes: suspected acute URTI, rash without fever, suspected acute LRTI, watery diarrhea, and skin, soft tissue, or bone abnormalities. The small number of alerts (n=26) indicated sufficient control of infectious disease spread at the 7 Austrian RCs. The signals triggered appropriate and prompt public health actions by the RCs’ health care staff and responsible public health authorities: investigations of alerts; exclusion of people suspected to be contagious from crowded activities, such as the RC refectory; contact tracing; scabies-related hygiene measures; intensified cleaning and disinfection in case of environmental contamination; and referral to specialized, secondary health care for adequate treatment. We identified suspected acute URTI as the predominant syndrome among the asylum seekers, which was similar to the experience with refugees arriving at the Greek-Turkish border [18] and among 51 RCs in Greece [19]. Our findings support previous experiences that asylum seekers, compared with the host population, mainly acquire infections with agents present among the host population, even though they are at higher risk due to many infection-favoring factors.

Interestingly, the second most frequently reported syndrome was rash without fever (scabies-like), for which over half of the signals (1032/1892, 54.49%) were reported in RC1. This was relatively higher than those reported among refugees in other countries, such as Greece [19]. Over half of the bed capacity of all RCs was provided by RC1 (1800/2840, 63.38%), which is considered as a large RC. According to the ECDC, exposure to crowded shelters increases the risk of the spread of lice, fleas, and mites [4]. Watery diarrhea accounted for less than 3% of reported syndromes among the RC residents, which is likely due to strict national food safety and control according to the Austrian Food Safety and Consumer Protection Act. Surprisingly, there was no norovirus outbreak in any of the RCs. A cluster of 21 cases of shigellosis occurred between July and November 2015 among refugees, mainly affecting transit centers in Austria. However, 3 cases of this cluster were identified in 3 of the 7 RCs, which indicates the sensitivity of the Austrian SbSS. In addition, completeness and timeliness, which were measured as system quality indicators, were high. Surprisingly, in RC5, acute URTI accounted for the smallest proportion across all RCs (39% vs 49% among the remaining RCs), whereas the proportion of LRTI was the highest in RC5. The opening of RC5 in December 2015 in the crowded urban setting of Vienna may explain the high rate of tracheobronchitis; 1 case also turned out to be pulmonary tuberculosis.

### Syndrome-Based Surveillance System Implementations in the European Union

Systematic data collection on asylum seekers’ health is limited in the EU [20]; therefore, there is a need to enhance national health surveillance systems [21], including being innovative in health surveillance methods to monitor the health of asylum seekers [22-24]. SbSS requires a near real-time automated data collection and analysis system [25]. Therefore, the Austrian SbSS was established in the form of a collaboration, and with a strong commitment, among different national authorities [13], including the Ministry of Interior, the RC health care staff, and the surveillance experts at the AGES. All signals and alerts were addressed on a daily basis through direct contact between the AGES and the focal RC SbSS. Sharing daily reports on syndromes with each RC and communicating signals and alerts immediately to both the focal RC SbSS and the Ministry of Health made appropriate action possible. We have observed that during the rollout of SbSS, the medical staff in nearby hospitals became more aware of the potential risk of communicable diseases among refugees. For example, in 1 of the RCs, located in Salzburg, a case of louse-borne relapsing fever was identified prior to the implementation of the SbSS (DS, unpublished data, 2016).

SbSS has been deployed in other high-income countries to supplement national public health surveillance systems. This includes during the 2006 heat wave in France [25], during the Olympic Games in each of Greece [26] and Italy [27], in Virginia, USA [28] during a national youth camp, in Sweden [29] using a Web-based query system for influenza, and in the United Kingdom [30] for the national telephone health helpline. In Austria, the AGES has introduced SbSS as a supplement to routine tuberculosis screening in selected migrant groups and to the routine national surveillance of 65 notifiable infectious diseases.

### Strengths and Limitations

The AGES had to face some limitations in operating the SbSS. The daily number of asylum seekers registered at the RCs was not reported in a consistent fashion. Thus, we could not calculate the incidence of syndromes among the asylum-seeking population. However, this is considered as a common limitation [16]. Of the 7 RCs, 5 were established on an ad hoc basis as temporary asylum seeker–hosting centers. In these ad hoc RCs, the availability of health care–providing staff was not consistent on a daily basis. This may have caused underdetection and underreporting of syndrome cases, particularly when asylum seekers were immediately transferred to the hospital. We analyzed the reported data in this study without comparison with a reference standard SbSS [30]; however, we need to acknowledge that, in general, SbSSs have a low specificity [31-33].

Strengths of the Austrian SbSS were, first, that data were collected in near real time [31] and that the largest RCs reported on a daily basis, which made the public health response time-efficient. Second, the SbSS had high sensitivity and practicability due to the use of easily ascertainable clinical signs without requiring laboratory testing [32,33]. Third, the Austrian SbSS could be deployed and implemented rapidly in the emergency situation of the most recent refugee crisis [10,22,31]. The SbSS in Austrian RCs proved to be highly eligible for identifying infectious diseases and detecting clusters among the asylum-seeking population.

### Conclusion

This was the first time that an SbSS was used in Austria for an increased number of incoming refugees seeking asylum. The SbSS was reliable at identifying and controlling the spread of infectious diseases among the asylum-seeking population from September 2015 onward.

## Abbreviations

AGES: Austrian Agency for Health and Food Safety
ECDC: European Centre for Disease Prevention and Control
EDC: expected daily case
ETH: expected threshold
EU: European Union
LRTI: lower respiratory tract infection
ODC: observed daily case
RC: reception center
SbSS: syndrome-based surveillance system
URTI: upper respiratory tract infection
